# Examining the impact of a province-wide physical education policy on secondary students’ physical activity as a natural experiment

**DOI:** 10.1186/s12966-017-0550-7

**Published:** 2017-07-19

**Authors:** Erin Hobin, Tannis Erickson, Melisa Comte, Fei Zuo, Saamir Pasha, Donna Murnaghan, Steve Manske, Catherine Casey, Jane Griffith, Jonathan McGavock

**Affiliations:** 1 Public Health Ontario, 480 University Ave, Suite 300, Toronto, ON M5R 1V2 Canada; 2 University of Waterloo, 200 University Ave W, Waterloo, ON N2L 3G1 Canada; 3 University of Toronto, 27 King’s College Circle, Toronto, ON M5S 1A2 Canada; 4 Children’s Hospital Research Institute of Manitoba, 513-715 McDermot Ave, Winnipeg, MB R3E 3P4 Canada; 5 University of Manitoba, 66 Chancellors Circle, Winnipeg, MB R3T 2N2 Canada; 6 Peel Public Health Unit, 150 Central Park Dr, Brampton, ON L6T 2T9 Canada; 7 Thompson Rivers University, 900 McGill Rd, Kamloops, BC V2C 0C8 Canada; 8 Propel Centre for Population Health Impact, 200 University Ave W, Waterloo, ON N2L 3G1 Canada; 9 Cancer Care Manitoba, 675 McDermot Ave, Winnipeg, MB R3E 0V9 Canada

**Keywords:** Physical activity, Adolescents, School-based policy, Physical education, Natural experiment

## Abstract

**Background:**

The purpose of this paper is to examine the impact of a province-wide physical education (PE) policy on secondary school students’ moderate to vigorous physical activity (MVPA).

**Methods:**

*Policy*: In fall 2008, Manitoba expanded a policy requiring a PE credit for students in grades 11 and 12 for the first time in Canada. The PE curriculum requires grades 11 and 12 students to complete a minimum of 55 h (50% of course hours) of MVPA (e.g., ≥30 min/day of MVPA on ≥5 days a week) during a 5-month semester to achieve the course credit.

*Study Designs*: A natural experimental study was designed using two sub-studies: 1) quasi-experimental controlled pre-post analysis of self-reported MVPA data obtained from census data in intervention and comparison [Prince Edward Island (PEI)] provinces in 2008 (*n* = 33,619 in Manitoba and *n* = 2258 in PEI) and 2012 (*n* = 41,169 in Manitoba and *n* = 4942 in PEI); and, 2) annual objectively measured MVPA in cohorts of secondary students in intervention (*n* = 447) and comparison (Alberta; *n* = 224) provinces over 4 years (2008 to 2012).

*Analysis*: In Study 1, two logistic regressions were conducted to model the odds that students accumulated: i) ≥30 min/day of MVPA, and ii) met Canada's national recommendation of ≥60 min/day of MVPA, in Manitoba versus PEI after adjusting for grade, sex, and BMI. In Study 2, a mixed effects model was used to assess students’ minutes of MVPA per day per semester in Manitoba and Alberta, adjusting for age, sex, BMI, school location and school SES.

**Results:**

In Study 1, no significant differences were observed in students achieving ≥30 (OR:1.13, 95% CI:0.92, 1.39) or ≥60 min/day of MVPA (OR:0.92, 95% CI: 0.78, 1.07) from baseline to follow-up between Manitoba and PEI. In Study 2, no significant policy effect on students’ MVPA trajectories from baseline to last follow-up were observed between Manitoba and Alberta overall (−1.52, 95% CI:-3.47, 0.42), or by covariates.

**Conclusions:**

The Manitoba policy mandating PE in grades 11 and 12 had no effect on student MVPA overall or by key student or school characteristics. However, the effect of the PE policy may be underestimated due to the use of a nonrandomized research design and lack of data assessing the extent of policy implementation across schools. Nevertheless, findings can provide evidence about policy features that may improve the PE policy in Manitoba and inform future PE policies in other jurisdictions.

## Background

Low levels of physical activity are associated with an increased risk for more than 25 chronic conditions including obesity, certain cancers and cardiovascular disease [1]. Despite its direct relationship with adverse health effects, global data indicate the majority of youth in 105 countries report inadequate physical activity levels [2]. Evidence suggests the proportion of under-active youth increases rapidly following puberty, reaching a pinnacle in adolescence where physical activity rates can decline as much as 85% by the age of 15 yrs. [3, 4]. Not surprisingly, the rates of several chronic diseases among youth, including type-2 diabetes and hypertension, are highest during adolescence [5–7]. This is a key concern as low levels of physical activity in adolescence predicts physical activity patterns in adulthood [8].

In Canada, surveillance data from objectively measured physical activity in 2007–2009 suggest that up to 93% of youth ages 6 to 19 do not achieve Canada’s recommendation of ≥60 min/day of moderate to vigorous physical activity (MVPA), and the percentages meeting this national physical activity recommendation decline with increasing age [9, 10] . Physical inactivity is associated with a cost between $2 and $6 billion to the Canadian economy [11]. Thus, public health experts are calling for strategies to increase physical activity rates among adolescents. Several provinces in Canada have responded to these calls and surveillance data by enacting school-based policies aimed at increasing student physical activity levels and improving health [12–14].

Physical activity is recognized as a complex behaviour that is influenced by several determinants, including individual, social, familial and environmental factors [15]. Adolescents frequently report that the major barriers to physical activity are environmental in nature including insufficient time, opportunity and access to resources [16–18]. Consequently, socio-ecological approaches are often cited as the best approach to elicit behaviour change that would lead to increased physical activity, especially in adolescents [19–21]. As schools provide access to almost all adolescents over extended periods of time they are considered important target environments for increasing adolescent physical activity levels [22]. Previous research supports this concept demonstrating that the school environment exerts a powerful influence on student behavior related to physical activity, obesity and smoking [23–26]. Accordingly, policies aimed at increasing physical activity frequently target the school environment; however, despite the widespread adoption of school-based physical activity policies, there is little data available describing their effectiveness.

Several investigator driven interventions designed to increase physical activity levels in youth have been evaluated in the school setting. Examples of school-based interventions include (1) novel curricula to increase knowledge of healthy living [27, 28], (2) environmental strategies to increase the capacity to alter behaviour [29], (3) the provision of activity breaks during non-physical education classes [30, 31] (reviewed in [32]), and (4) enhanced physical education (PE) curriculum that includes providing free choice of activities [27, 33–37]. Collectively, the results of these interventions support the concept that school-based interventions are efficacious for increasing physical activity among youth [38]. With the odd exception [39], the effectiveness of policies mandating these approaches remains uncertain as few school-based physical activity interventions have been widely disseminated and few state- or province-wide policies have been evaluated. The gap in our understanding of the effectiveness of these approaches is particularly true for adolescents, who are the least physically active [9] and at the greatest risk of developing cardiometabolic complications [40] and are rarely the target audience for school-based policies. Lastly, with the exception of sex, the impact of physical activity interventions on population sub-groups remains largely unknown. With this in mind, the objectives of this research were to use a large natural experiment to examine the impact of a novel province-wide physical education (PE) policy on secondary school students’ physical activity, and to assess if the PE policy had a differential impact on students’ physical activity trajectories by student and school characteristics. We hypothesized that the PE policy would increase student MVPA overall, and especially among students attending schools in lower SES neighbourhoods who tend to participate in less MVPA and have fewer opportunities for physical activity.

## Methods

### The PE policy

**Fig. 1:**
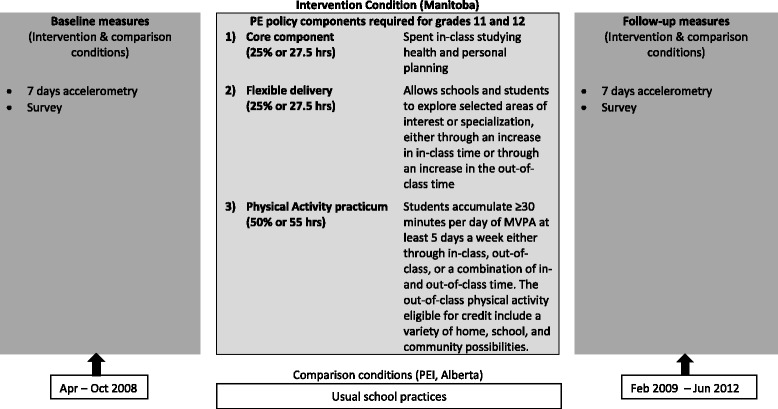
PE policy components and timing of delivery

In the fall of 2008, the province of Manitoba enacted a policy, the first of its kind in Canada, extending secondary school graduation requirements from two to four PE credits [41]. The policy added PE curriculum in all four years of secondary school, mandating PE credits for the completion of grades 11 and 12. The PE curriculum for grades 11 and 12 students requires a total of 110 credit hours divided into three components: 1) core; 2) flexible delivery; and, 3) PA practicum (Fig. 1). The core component is a minimum 25% (~30 h) spent in-class studying health and personal planning. The flexible delivery component allows schools and students to choose up to 25% of the credit hours exploring selected areas of interest or specialization, either through an increase in the in-class time, or through an increase in the out-of-class time, depending on local resources and needs. The physical activity practicum requires a minimum 50% (55 h) focused on participation in physical activity. The 55-h time frame for the practical physical activity component was established based on the expectation that students need to accumulate ≥30 min/day of MVPA at least 5 days a week to achieve the course credit (i.e., 50% of the minimum 60 min/day of MVPA identified in Canada’s physical activity guidelines). Students can achieve the physical activity practicum hours through in-class, out-of-class, or a combination of in- and out-of-class time. The out-of-class physical activities eligible for credit include a wide variety of home, school and community possibilities tailored to meet students’ needs, interests, and opportunities.

### Study design

With the understanding that the PE policy was being implemented in Manitoba in the 2008/2009 school year, a natural experiment with comparison provinces was designed using two complementary studies: 1) census self-reported physical activity data obtained from surveillance studies, and 2) objectively measured physical activity in selected cohorts of students before and after the policy was implemented (Fig. 1). As part of the Manitoba Increasing Physical Activity in Secondary Students (MIPASS) study, baseline and follow-up data were collected among secondary students in the intervention province of Manitoba that mandates PE in grades 11 and 12, as well as in two Canadian provinces [Prince Edward Island (PEI) and Alberta] that do not mandate PE in grades 11 and 12. Manitoba and Alberta are located in the prairies in Western Canada with populations of 1.3 and 4.1 million, respectively. PEI is the smallest province in Canada, with a population of approximately 146,000, and is located in Eastern Canada. Each of these three Canadian provinces has a moderate climate and experiences four seasons annually. Study 1 examines the impact of the PE policy on student physical activity using self-reported physical activity surveillance data. These data were collected during two cycles (2007–08, 2012–13) of cross-sectional, machine-scanned paper-based surveys among a census of grades 9 to 12 students in Manitoba (Youth Health Survey) [42] and in the comparison province, PEI (SHAPES-PEI) [43]. Due to the time gap between the two cross-sectional survey data collections, different students were assessed at baseline and follow-up. The survey tools in Manitoba and PEI self-reported on key demographic variables and included measures that were previously validated for assessing adolescent MVPA [44]. Study 2 uses accelerometer data collected longitudinally before the PE policy in 2008 (baseline), and yearly for three years after the implementation of the PE policy (follow-up) from a small cohort of students in Manitoba (intervention) and in Alberta (comparison) to report precise changes in physical activity by student and school characteristics. The accelerometer data collected among secondary students in Manitoba were previously examined to determine the physical activity trajectories of students throughout secondary school [45]; however, this study lacked a comparison condition. The current study has the advantage of including both repeated cross-sectional and longitudinal data sources with comparison groups to examine the impact of the PE policy on student physical activity.

### Study sample

In Study 1, a census of self-reported physical activity and its determinants were conducted from 2006 to 2008 among 232 schools with grades 9 to 12 (33,619 students) in the intervention province of Manitoba. Using the same tool and similar protocols, follow-up data were then collected in Manitoba during the 2012–2013 school year among a census of 274 schools (41,169 students). A census was also conducted in 22 schools (2258 students) in the comparison province of PEI in the 2007–2008 school year using a similar survey instrument and protocols with identical measures assessing MVPA, and again in 2012–2013 among 24 schools (4942 students). Student participation was voluntary and consent was obtained through active-information, passive-permission procedures. The procedures were approved by the Human Research Ethics Board at the University of Manitoba (Ref #H2014:396) and the University of PEI (Ref # 1002929, #6004948).

**Fig. 2:**
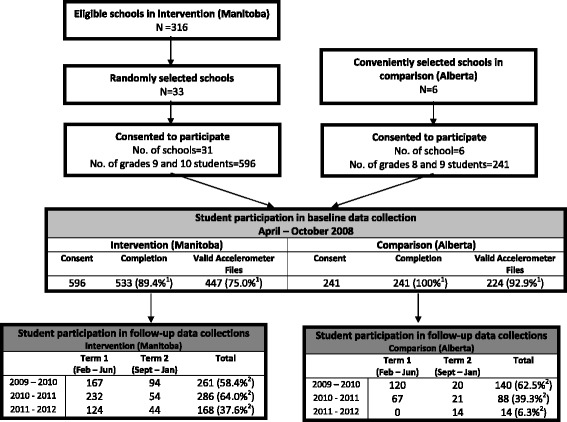
Flowchart describing progress of participants through Study 2 (accelerometer). ^1^Denominator is number of students with parental consent in Intervention (Manitoba, *n* = 596) and Comparison (Alberta, *n* = 241). ^2^Denominator is number of students that participated in baseline data collection in Intervention (Manitoba, *n* = 447) or Comparison (Alberta, *n* = 224).

In Study 2, between 2008 and 2012, our research team measured physical activity levels objectively over a period of 7 days each year using accelerometry in cohorts of students in Manitoba (intervention) and in Alberta (comparison). Prior to baseline data collection, all secondary schools in the province of Manitoba were placed on a list to be contacted for participation. Randomization was blocked to ensure that 40% of schools selected were from rural areas to adequately represent the province of Manitoba. Entry criteria for participating in the cohort study in Manitoba included a minimum of 100 students offering grades 9 through 12. As shown in Fig. 2, among the 316 possible schools in the province that satisfied entry criteria, 33 were randomly selected. In total, 31 of the 33 schools agreed to participate and assigned a lead teacher or administrator to facilitate data collection. Lead teachers within each school selected a grade 9 or 10 PE classroom to approach for participation. The study was explained to students and 596 decided to participate in the prospective longitudinal tracking of physical activity with accelerometers through their secondary school tenure. However, of the 596 grade 9 and 10 students recruited, 63 (10.6%) students did not return the accelerometers at baseline, 65 students did not meet minimum accelerometer wear time requirements at baseline, and 21 students had an extreme MVPA baseline measurement (baseline values beyond the 95% percentile, i.e., ≥122 min/day of MVPA; *n* = 21) and were removed from analysis, leading to a final sample size of 447 (75.0%) students. These students were followed up once a year until January 2012 or grade 12, whichever came first. For the comparison condition, two of the investigators were involved in an on-going prospective cohort study of physical activity and risk factors for type 2 diabetes in youth in the province of Alberta that does not mandate PE credits in grades 11 and 12. This school-based study included 241 students in grades 8 and 9 in 2008 from six middle or high schools selected for convenience, but reflecting urban and rural settings. Accelerometer data were collected from the student cohorts in Manitoba and Alberta every 10 to 13 months for 3 years after the initial visit. To enhance student retention, we offered the students a $5 honorarium per day, up to $25 for the week, for each day that they wore their accelerometer for a minimum of 8 h. Students received their money at the end of the data collection period along with a catered lunch for all the students who participated in the study that year. At the end of the data collection for that year, students received a feedback form with their individual data along with the average province wide data. All parents and students provided written informed consent in accordance with Declaration of Helsinki. The procedures were approved by the Human Research Ethics Board at the University of Manitoba (Ref #H2014:396 and #E2008:042) and the University of Alberta (Ref #5064).

### Outcome measures

Study 1: Using the survey data, three outcome measures were used to assess student physical activity:

1. accumulating the PE policy requirement of ≥30 min/day of MVPA on at least 5 days in a week,
2. accumulating the Canadian physical activity recommendation of ≥60 min/day of MVPA, and
3. kilocalories per kilogram per day (KKD) which is an estimate of how much energy a person has expended in a day.

To calculate mean minutes per day of MVPA, each student’s responses to the items “Mark how many minutes of moderate physical activity you did on each of the last 7 days” and “Mark how many minutes of hard physical activity you did on each of the last 7 days” were summed and divided by 7 days. Responses were provided by indicating the number of hours (0–4) and 15-min increments (0–45 min) that each type of physical activity was performed for each day of the previous week. Student physical activity levels were based on calculated KKD (kilocalories/kg of body weight/day). Mean KKD expended in hard and moderate physical activity were calculated as: KKD = [(hours of hard physical activity in past 7 days × 6 MET) + (hours of moderate physical activity in past 7 day × 3 MET)]/7 days. Using these KKD data, students’ physical activity levels were categorized as: Inactive = 0 ≤ KKD < 3; Moderately Active = 3 ≤ KKD ≤ 8; and, Active = KKD >8.

Study 2: The primary outcome measure was daily minutes of MVPA. Raw physical activity counts were collected in 30 s epochs, converted into minutes of activity at specific intensities using a specially-designed software program (KineSoft, Saskatoon, SK.) [46, 47]. Raw-count cut-points for sedentary behaviour (0–49 cpm), light (50–749 cpm), moderate (750–3249 cpm) and vigorous (>3250 cpm) minutes of physical activity were based on validation studies in youth using the Actical device [48]. Sedentary time was calculated as part of the inclusion criteria for estimating physical activity. Inclusion criteria for retaining data in the final analysis were a minimum of 3 days of wear with data available and at least 480 registered minutes/day as previously described [49]. Sequences of consecutive zero counts equal or greater to 60 min were deemed non-wear and excluded from analyses.

### Potential student- and school-level confounding variables

Students participating in the survey and accelerometry studies in intervention and comparison provinces were asked to report their age, grade, sex, height, and weight at each data collection time point. Age- and sex-adjusted body mass index (BMI) cut-points derived from the WHO growth charts were used to classify students’ weight status. As the prevalence of underweight was very low, this category was merged with healthy weight. School location and neighbourhood socioeconomic factors were examined as potential confounding variables in the cohort sample. These factors were identified by linking school postal codes to the 2006 Canadian census data and the Institut national de santé publique due Quebec (INSPQ) material deprivation index at the dissemination area level, which corresponds to geographical areas of 400 to 700 people [50]. School locations were classified as urban or rural, with rural defined as having a 2006 census population of less than 10,000. School neighbourhoods were classified as having high or low socioeconomic status (SES), as measured by the validated material deprivation index [50].

### Statistical analyses

Descriptive statistics of continuous and categorical variables were performed at baseline across the study population and provinces for the survey and accelerometer data. For Study 1, two separate logistic regressions were conducted to model the odds that students accumulated ≥30 and ≥60 min/day of MVPA on at least 5 days a week. Sensitivity analyses were undertaken to assess differences in the proportion of students accumulating ≥30 and ≥60 min/day of MVPA on at least 4 days a week compared to at least 5 days a week, as more students provided complete MVPA data for at least 4 versus 5 days a week. Plus, a multinomial logistic regression was used to model the odds that students were active, and moderately active, relative to inactive, respectively. The final models included study condition (Comparison vs Intervention), time, an interaction term between study condition and time, as well as grade, sex, and BMI to examine whether students exposed to the PE policy were more likely to be active than students in the comparison condition not exposed to the PE policy at follow-up compared to baseline while adjusting for demographic covariates.

In Study 2, mixed effects models were used to account for school-level clustering and within-student correlation. First, an intercept-only mixed effects model was used to examine the amount of variability in measurements of MVPA (level 1) due to students (level 2) and schools (level 3), respectively (results not shown). Next, a mixed effects model including a semester variable (T0-T6) was used to assess students’ MVPA at baseline and change in MVPA from baseline to the last follow-up (results not shown). Subsequently, six separate mixed effects models were conducted, each adjusting for semester, one of the following covariates (condition, age, sex, BMI, school location, and school neighbourhood SES), and its interaction with semester. Lastly, a multivariable growth curve model was conducted adjusting for the aforementioned covariates, as well as their interactions with semester. This technique was applied because it can accommodate individuals who have unequal spacing in data collection and missing data in covariates and across time. Significant interactions between covariates and semester were further examined in three-way interactions with condition. All analyses were conducted using SAS v 9.3 software (SAS Institute, Inc., Cary, NC).

## Results

### Study 1

#### Participant characteristics

The characteristics of student participants by study are provided in Table 1. Overall, student participants had a mean age of 15.8 ± 0.71 in PEI and 15.7 ± 0.82 years in Manitoba, and were almost equally distributed between males and females and across grades 9 through 12. The majority of students were categorized as having a healthy weight in both comparison and intervention conditions. Significant differences between comparison and intervention conditions were detected for age, grade, sex, and BMI status, and were controlled for in the regression analyses.

**Table 1:** Baseline characteristics by study

Study 1: Survey data	Comparison (PEI) (*n* = 2258)		Intervention (Manitoba) (*n* = 33,619)		*P* value
Age (years), mean ± SD	15.8	0.71	15.7	0.82	<.0001*
	n	%	n	%	
Grade					
9	576	(25.5)	9070	(27.0)	0.0005^†^
10	591	(26.2)	9216	(27.4)	
11	543	(24.0)	8438	(25.1)	
12	548	(24.3)	6895	(20.5)	
Sex					
Female	1053	(46.6)	16,846	(50.1)	<.0001^†^
Male	1094	(48.4)	16,773	(49.9)	
Missing	111	(4.9)	-	-	
BMI status					
Healthy weight	1252	(55.4)	21,505	(64.0)	<.0001^†^
Overweight or obese	468	(20.7)	7107	(21.1)	
Missing	538	(23.8)	5007	(14.9)	
Study 2: Accelerometer data	Comparison (Alberta) (*n* = 224)		Intervention (Manitoba) (*n* = 447)		*P* value
Age (years), mean ± SD	13.5	0.71	15.2	0.82	<.0001*
	n	%	n	%	
Sex					
Female	133	(59.4)	239	(54.0)	0.1828^†^
Male	91	(40.6)	204	(46.1)	
BMI status					
Healthy weight	175	(78.1)	228	(51.0)	<.0001^†^
Overweight or obese	49	(21.9)	61	(13.7)	
Missing	-	-	158	(35.4)	
School location					0.0181^†^
Urban	137	(61.2)	314	(70.3)	
Rural	87	(38.8)	133	(29.8)	
School Socioeconomic status					<.0001^†^
High	186	(83.0)	139	(31.1)	
Low	38	(17.0)	308	(68.9)	

Note: For accelerometer data, missing categories that constitute less than 5% of the sample were omitted from the table. Therefore, counts may not add up to their respective totals

*Student’s t-test was used to compare across study condition

^†^Chi-square test was used to compare across province

### Effect of the PE policy on students’ physical activity

**Fig. 3:**
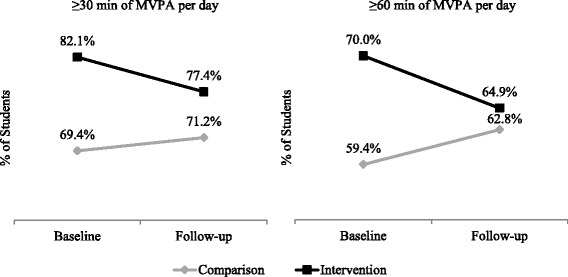
Results from Study 1 (survey): Proportion of students accumulating ≥30 and ≥60 min of MVPA per day by condition

The findings of sensitivity analysis examining differences in considering 4 or 5 days a week of MVPA confirmed applying a cut-off of 4 days in a week did not significantly alter results; thus results applying a cut-off of 4 days a week are presented. The proportion of students who met the PE policy requirement of ≥30 min/day of MVPA was 69.4% at baseline and 71.2% at follow-up in the comparison condition, and 82.1% at baseline and 77.4% at follow-up in the intervention condition. Similarly, the proportion of students who met the Canadian physical activity recommendation of ≥60 min/day of MVPA was 59.4% at baseline and 61.8% at follow-up in the comparison condition, and 70.0% at baseline and 64.9% at follow-up in the intervention condition. The proportion of students who were categorized as active, moderately, active, and inactive according to their KKD estimate are shown in Fig. 3.

The results of the regression models examining differences in student physical activity between baseline and follow-up by study condition are provided in Table 2. Students categorized as active were significantly more likely to be male (OR = 2.26, 95% CI:2.10, 2.43), and less likely to be in grades 10 through 12 as compared to grade 9, and overweight and obese relative to a healthy weight (OR = 0.63, 95% CI:0.47, 0.85). No significant differences were observed in the proportion of students achieving ≥30 (OR:1.13, 95% CI:0.92,1.39) or ≥60 min/day of MVPA (OR:0.92, 95% CI: 0.78, 1.07) from baseline to follow-up between conditions. No differences were observed in the proportion of students being categorized as moderately active versus inactive (OR = 0.82, 95% CI: 0.61, 1.11). However, the results indicate there was a significant difference from baseline to follow-up in the students categorized as “active versus inactive” between study conditions, where students in the intervention condition were significantly less likely to be categorized as active at follow-up than at baseline relative to students in the comparison condition (OR = 0.63, 95% CI:0.47, 0.85).

**Table 2:** Study 1: Separate models examining estimated odds ratios for self-reported physical activity outcomes adjusting for student characteristics

Survey Data	Model 1–30 min/day		Model 2–60 min/day		Model 3 – KKD			
					*Active* vs *Inactive*		*Moderately Active* vs *Inactive*	
	Fixed effect (95% CI)	*P* value	Fixed effect (95% CI)	*P* value	Fixed effect (95% CI)	*P* value	Fixed effect (95% CI)	*P* value
Study Condition								
Comparison (PEI)	Referent	<.0001	Referent	<.0001	Referent	0.23	Referent	0.32
Intervention (Manitoba)	2.24 (1.88, 2.66)		1.32 (1.16, 1.50)		1.16 (0.91,1.48)		1.13 (0.89, 1.45)	
Time								
Baseline	Referent		Referent		Referent		Referent	
Follow-up	0.79 (0.65, 0.95)	0.01	0.95 (0.82, 1.10)	0.49	1.13 (0.84, 1.51)	0.41	1.15 (0.86, 1.52)	0.35
Condition*Time Interaction	1.13 (0.92, 1.39)	0.24	0.92 (0.78, 1.07)	0.26	0.63 (0.47, 0.85)	0.0021	0.82 (0.61, 1.11)	0.20
Grade								
9	Referent		Referent		Referent		Referent	
10	0.91 (0.82, 1.00)	0.05	0.91 (0.86, 0.97)	0.0016	0.84 (0.75, 0.93)	0.0008	0.92 (0.82, 1.02)	0.12
11	0.75 (0.68, 0.82)	<.0001	0.73 (0.69, 0.77)	<.0001	0.58 (0.52, 0.64)	<.0001	0.81 (0.73, 0.90)	<.0001
12	0.66 (0.60, 0.73)	<.0001	0.68 (0.64, 0.72)	<.0001	0.52 (0.47, 0.57)	<.0001	0.80 (0.72, 0.89)	<.0001
Sex								
Female	Referent	<.0001	Referent	< .0001	Referent	< .0001	Referent	<.0001
Male	1.87 (1.74, 2.00)		1.88 (1.80, 1.95)		2.26 (2.10, 2.43)		1.29 (1.20, 1.39)	
BMI status								
Healthy weight	Referent	0.0007	Referent	0.0006	Referent	< .0001	Referent	0.20
Overweight or obese	0.88 (0.81, 0.95)		0.92 (0.88, 0.97)		0.85 (0.78, 0.92)		0.95 (0.87, 1.03)	

Note: Fixed effects of intercepts for each model are not shown. 95% confidence intervals are shown in parentheses. Referent categories are identified as “Referent”

### Study 2

#### Participant characteristics

Baseline characteristics are shown in Table 1. The participating students from both comparison and intervention conditions were equally distributed between males and females. However, there were significant differences between study conditions in participant age and BMI status, school neighbourhood location, and particularly in school SES, where 17.0% versus 68.9% of schools were categorized as lower SES in comparison and intervention conditions, respectively.

#### Baseline physical activity

Objectively measured physical activity at baseline and follow-up periods is presented in Table 3. Each student contributed an average of 2.4 ± 1.0 assessments over the study duration. At baseline, students in the comparison condition accumulated 53.0 ± 21.9 min/day of MVPA as compared to 58.3 ± 24.0 min/day of MVPA in the intervention condition (*p* = 0.004). Greater than 85% of the students accumulated the PE policy requirement of ≥30 min/day of MVPA at baseline in both study conditions (*p* = 0.46), while 33.9% in the comparison condition and 44.5% in the intervention condition met the Canadian physical activity recommendation of ≥60 min/day of MVPA at baseline (*p* = 0.009). The growth curve model revealed that MVPA was different between sexes and across school neighbourhood SES (Table 4). Compared to males, females accumulated 11.1 fewer minutes/day of MVPA at baseline (*p*< .0001). Students attending schools located in neighbourhoods of low SES accumulated an average of 10.2 fewer minutes/day of MVPA at baseline compared to students attending schools in neighbourhoods of high SES (*p* = 0.005).

**Table 3:** Study 2: Objectively measured physical activity at baseline and follow-up by study condition and total sample

	Comparison (Alberta) (*n*=224)		Intervention (Manitoba) (*n* =447)		Total (*n* = 671)		
Accelerometer data	*n*	(%)	*n*	(%)	*n*	(%)	*P* value
Students with valid MVPA at time point							
T0: Pre-policy	224	(100.0)	447	(100.0)	671	(100.0)	N.A.
T1: February 2009 – June 2009	120	(53.6)	167	(37.4)	287		N.A.
T2: September 2009 – January 2010	20	(8.9)	94	(21.0)	114	(42.8)	N.A.
T3: February 2010 – June 2010	67	(29.9)	232	(51.9)	299	(17.0)	N.A.
T4 : September 2010 – January 2011	21	(9.4)	54	(12.1)	75	(44.6)	N.A.
T5 : February 2011 – June 2011	0	(0)	124	(27.7)	124	(11.2)	N.A.
T6 : September 2011 – January 2012	14	(6.3)	44	(9.8)	58	(18.5) (8.6)	N.A.
Times points contributed/ student, mean ± SD	2.1	0.8	2.6	1.1	2.4	1.0	<.0001^*^
Baseline MVPA							
Students with baseline data	224	(100.0)	447	(100.0)	671	(100.0)	N.A.
Minutes/day, mean ± SD	53.0	21.9	58.3	24.0	56.5		0.0039^†^
Accumulated ≥ 30 min/day	194	(86.6)	396	(88.6)	590	23.4	
Accumulated ≥ 60 min/day	76	(33.9)	199	(44.5)	275	(87.9) (41.0)	0.4571^†^ 0.0085^†^
MVPA at students’ last follow-up							
Students with ≥ 1 follow up data	165	(73.7)	340	(76.1)	505	(75.3)	0.4965^†^
Minutes/day, mean ± SD	49.4	22.2	44.1	23.7	45.8	23.3	0.0143^*^
Accumulated ≥ 30 min/day (% with ≥ 1 follow up)	131	(79.4)	231	(67.9)	362	(71.7)	0.0074^†^
Accumulated ≥ 60 min/day (% with ≥ 1 follow up)	48	(29.1)	71	(20.9)	119	(23.6)	0.0415^†^
MVPA change from baseline to last follow up							
Change (min/day), mean ± SD	-3.7	25.3	-13.4	26.2	-10.3	26.3	<.0001^*^
Mean percentage change (%), mean ± SD	14.2	130.5	-15.9	49.6	-6.1	86.0	0.0047^*^

N.A. denotes not applicable

*Student’s t-test was used to compare across study condition

^†^Chi-square test was used to compare across study condition

*P-value* = comparison of comparison and intervention cohorts

**Table 4:** Multivariable mixed effects model examining estimated effect on students’ baseline MVPA and MVPA trajectories adjusting for study condition, student characteristics, and school neighbourhood characteristics

Accelerometer data	Fixed effect (95% CI)	*P* value
Estimated effect on baseline MVPA (minutes/day)		
Study condition		
Comparison (Alberta)	Referent	0.0799
Intervention (Manitoba)	8.23 (−0.98, 17.43)	
Baseline age	0.58 (−1.83, 2.99)	0.6366
Sex		
Male	Referent	<.0001
Female	−11.09 (−14.16, −8.02)	
BMI status		
Healthy weight	Referent	
Overweight or obese	0.02 (−4.07, 4.12)	0.9898
Missing	−0.57 (−5.33, 4.20)	0.8150
School rural/urban		
Urban	Referent	0.5982
Rural	−1.85 (−8.72, 5.03)	
School socioeconomic status		
High	Referent	0.0048
Low	−10.24 (−17.35, −3.14)	
Estimated effect on MVPA trajectories (minutes/day per semester)		
Study condition		
Comparison	Referent	0.1243
Intervention	−1.52 (−3.47, 0.42)	
Baseline age	−0.62 (−1.38, 0.15)	0.1143
Sex		
Male	Referent	0.2846
Female	0.55 (−0.46, 1.57)	
BMI status		
Healthy weight	Referent	
Overweight or obese	−0.40 (−1.83, 1.04)	0.5858
Missing	−0.18 (−1.42, 1.07)	0.7792
School rural/urban		
Urban	Referent	0.4673
Rural	0.47 (−0.80, 1.74)	
School socioeconomic status		
High	Referent	0.0013
Low	2.13 (0.83, 3.43)	

Note: Fixed effects of intercepts for each model are not shown. 95% confidence intervals are shown in parentheses. Referent categories are identified as “Referent”

#### Effect of the PE policy on students’ physical activity trajectories and student- and school-level predictors

**Fig. 4:**
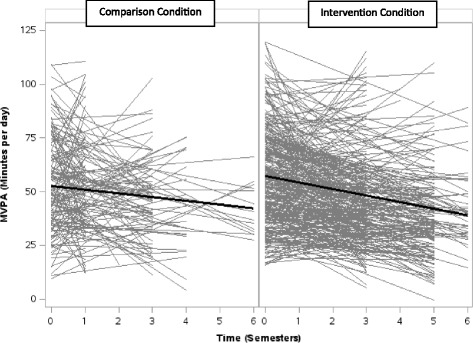
Results from Study 2 (accelerometer): Student MVPA trajectories from April 2008 to January 2012, by study condition. *Linear* regressions of MVPA (minutes per day) across semesters for each student with baseline and at least 1 valid follow-up measurement, by study condition, are shown in *gray*. *Linear growth curves* showing averaged baseline MVPA and averaged rate of change in MVPA among students, by study condition, are shown in *black*

**Fig. 5:**
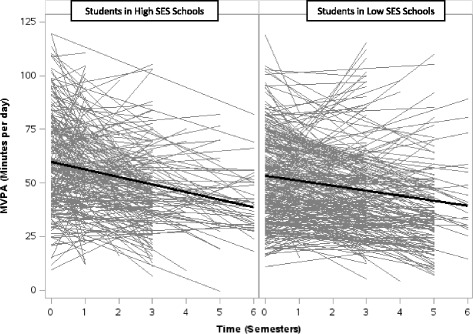
Results from Study 2 (accelerometer): Student MVPA trajectories from April 2008 to January 2012, by socioeconomic status. *Linear* regressions of MVPA (minutes per day) across semesters for each student with baseline and at least 1 valid follow-up measurement, by school neighbourhood SES, are shown in *gray*. *Linear growth curves* showing averaged baseline MVPA and averaged rate of change in MVPA among students, by socioeconomic status, are shown in *black*

Among the students in comparison (*n* = 165) and intervention (*n* = 340) conditions who provided baseline and at least one valid follow-up observation (75.3% overall, Table 4), an average of 49.4 ± 22.2 and 44.1 ± 23.7 min/day of MVPA was accumulated at the last follow-up, respectively. This represents a decrease of 3.7 ± 25.3 min/day of MVPA from baseline to last follow-up in the comparison condition and a decrease of 13.4 ± 26.2 min/day of MVPA in the intervention condition (*p*< .0001, Fig. 4). The proportion of students who accumulated ≥30 min/day also fell to 79.4% and 67.9% in comparison and intervention conditions, respectively (*p* = 0.007). Similarly, the proportion of students who accumulated ≥60 min/day of MVPA decreased to 29.1% in the comparison and 20.9% in the intervention condition (*p* = 0.04). In the multivariable linear growth curve model (Table 4), there were no significant differences in students’ physical activity trajectories between baseline and last follow-up by study condition (*p* = 0.12; Table 4). However, the association of school neighbourhood SES with students’ physical activity trajectories was statistically significant. Compared with students who attended schools located in neighbourhoods with high SES, the adjusted physical activity trajectories of students attending schools located in neighbourhoods with low SES declined less steeply, differing by an average of 2.1 min/day of MVPA across each semester (*p* = 0.0013, Fig. 5). The difference in the adjusted physical activity trajectories across baseline age, sex, BMI status, and school location was not statistically significant. Sensitivity analyses examining a three-way interaction to test if students’ physical activity trajectories in comparison and intervention conditions significantly differed by school neighbourhood SES were not significant (*p* = 0.20).

## Discussion

To our knowledge, this is the first study to examine the effect of a province-wide secondary school PE policy intervention on physical activity levels among adolescents as a natural experiment. The combination of survey and accelerometer data collected prior to and following the PE policy strengthens the conclusion that the PE policy did not significantly improve students’ physical activity throughout their secondary school tenure. The data suggest that school PE-based policies may not be sufficient on their own to attenuate age-related declines in physical activity among older adolescents, yet provide evidence to improve the current PE policy, and inform future policies and natural experimental studies of these policies.

A systematic review of randomized trials of school-based physical activity interventions reveals that these approaches can have modest effects on student physical activity rates and duration [43]; however, few studies target adolescents, especially older adolescents, and results indicate little effects. Common features of school-based interventions that successfully increase physical activity include PE curricula development, offering free choice of activities, community physical activity links, and multicomponent, flexible delivery models [27–37]. The PE policy examined in this paper includes these common features of successful physical activity interventions, yet the findings of this evaluation suggest that when school-based efforts are scaled up they may be less effective. There are several potential reasons for the discrepant results between randomized trials and the current natural experimental study of a regional policy. It is possible that that the dose of MVPA mandated under the PE policy is insufficient. Under the PE policy studied here, students are required to accumulate ≥30 min/day of MVPA on at least 5 days a week to achieve the course credit [41]. Results of both the survey and accelerometer data indicate the majority of student participants achieve this target at both baseline and follow-up regardless of being exposed to the PE policy, indicating that a PE policy is not necessary to achieve this level of activity. A much smaller proportion of students achieved ≥60 min/day of MVPA in the comparison and intervention conditions, particularly at follow-up; on average, the students required an additional 11 to 16 min/day of MVPA to meet this threshold. Thus, increasing the dose of policy mandated physical activity from ≥30 to ≥60 min/day of MVPA may better support student physical activity.

It is also possible that the lack of intervention effect of the PE policy is due to issues with implementation. Although the PE policy in Manitoba is mandatory for all publicly funded secondary schools across the province, little data were collected by our MIPASS team to evaluate the extent to which the PE policy was delivered in schools and received by all grades 11 and 12 students as intended. Findings from unpublished qualitative interviews conducted by the MIPASS team 1-year following policy implementation with PE teachers in 12 of the 31 schools participating in Study 2 suggest that there was a high level of commitment and support for the PE policy, yet the required time and demanding workload on PE teachers as well as the lack of facilities in schools were perceived as barriers to implementing the PE policy. It is not possible from the MIPASS evaluation however, to determine the variation in the level of implementation of the PE policy across schools or what proportion of students successfully achieved their PE course credits in grades 11 and 12. Given the flexible nature of this PE policy in Manitoba, assessing intervention fidelity and reach, as well as the various delivery models applied by schools to meet their schools’ and students’ needs, priorities, and preferences would provide important evidence to inform the current PE policy and future school-based PE interventions.

Another alternative explanation for the null effect of the PE policy is the baseline differences in physical activity levels between the intervention and comparison conditions in both Studies 1 and 2. In Studies 1 and 2, higher proportions of participants met the ≥30 and ≥60 min/day of MVPA recommendations in the intervention condition relative to the comparison condition, and these differences in the proportions of students meeting the MVPA recommendations are potentially caused by or at least not controlled for in the non-randomized design applied in this research. Despite the differences in student MVPA at baseline, however, the trends from baseline to follow-up across Studies 1 and 2 are congruent in that they signal a lack of intervention effect on student MVPA. For example, as shown in Fig. 3, the proportion of students meeting the ≥30 and ≥60 min recommendations in Study 1 decreases from baseline to follow-up in the intervention condition, and slightly increases in the comparison condition, with qualitatively similar proportions of students in both conditions meeting the MVPA cut-offs at follow-up. In Study 2, the proportions of students meeting the ≥30 and ≥60 min recommendations decrease in both the intervention and comparison conditions, with a greater decline in minutes per day of MVPA among students exposed to the policy relative to the comparison condition. Taken together, the consistency in these results increases the credibility of the findings that the PE policy did not improve student MVPA.

Lastly, previous evidence indicates that youth living in lower SES neighbourhoods are less active and at a greater risk for chronic disease than youth living in higher SES neighbourhoods [51–53]. Adolescents from disadvantaged backgrounds also tend to experience a steeper decline in physical activity [54]. Two recent studies, one among elementary school students in Canada and one among secondary school students in Australia, revealed that comprehensive school interventions can eliminate social inequities in physical activity among youth [55, 56]. Results of the current study indicate that, compared to schools located in high SES neighbourhoods, students attending schools in low SES neighbourhoods had lower levels of physical activity in both intervention and comparison conditions throughout the 4-year study; yet, in contrast to previous research, the students in low SES neighbourhoods experienced a 2.1 min per day per semester attenuation in the decline in physical activity over the course of their secondary school tenure. This attenuation in the decline in physical activity cannot be attributed to the PE policy, however, as there was no significant difference between students’ physical activity in low SES schools in intervention and comparison conditions before and after the PE policy. Instead, it is possible that this attenuated decline in physical activity may be related to a regression to the mean phenomenon, whereby students’ physical activity levels in low SES neighbourhoods is regressing over time towards the population average. As evidence for physical activity interventions among students of varying social disadvantage are scarce, more research examining the differential impact of PE policies on the physical activity levels of specific population sub-groups is urgently needed to better target interventions for those with greatest need.

Previous evaluations of school-based interventions aimed at increasing physical activity were limited in that many did not require follow-up data over the longer term, use objective measures of physical activity, target adolescents (i.e., ages ~14–18 years), and include subgroup analysis to examine differences in physical activity by sex, age, and SES [38]. The current natural experimental study builds on this work in several ways. First, relying on repeated cross-sectional census data and longitudinal measured physical activity data from large cohorts over a 4-year period provided a unique opportunity to assess the longer-term impact of the policy at various levels. Second, the addition of parallel data from provinces that did not enact a PE policy allowed control for any potential confounding effects of changes in physical activity due to maturation threats or seasonal changes. Finally, by focusing on adolescents, the period of childhood development when decrements in physical activity are the highest, this study was able to assess a policy aimed at the population with the highest risk of physical inactivity. The robust nature of this evaluation and focus on a high risk group strengthen the notion that school-based policies designed to increase physical activity need to be more intensive in terms of the required minutes per day of MVPA, support physical activity both during school hours and outside school hours, strengthen community involvement, and involve ongoing student input to be more effective.

### Limitations

There are a number of limitations in this study. First, in addition to the baseline differences in physical activity levels described above, the student participants in the comparison and intervention conditions also differed on a number of factors other than being exposed to the PE policy. As a result, differences in student physical activity cannot be attributed to any one factor. Applying a quasi-experimental design that uses both pretests and comparison groups allows us to assess the direction and magnitude of initial group differences on key variables, as well as control for these differences in the demographic profiles between sites. Nevertheless, it should be noted that the effect of the PE policy may have been underestimated since the cohort participants providing accelerometer data in the comparison group in Study 2 were significantly younger relative to the intervention group. This may be critical as the physical activity levels of youth tend to decline significantly over the course of later adolescence [3, 4, 9]. Matching comparison and intervention samples on key characteristics known to associate with MVPA, such as sex, age, and SES should be considered in future evaluations. Second, a significant proportion of the invited schools in comparison and intervention conditions did not participate in the survey data collections at baseline and follow-up, and there is no data to provide information about the characteristics of students that did not participate in the surveys. Moreover, there may be some bias in the accelerometer data due to selective attrition. The samples in both the comparison and intervention conditions were conveniently sampled and may in part contribute to the relatively large proportion of participants in the current study who were at a healthy weight and who met the Canadian physical activity recommendation of ≥60 min/day of MVPA at both baseline and last follow-up measures compared to the nationally representative sample in the Canadian Health Measures Survey [9, 10]. This sampling strategy may limit the generalizability of the findings; however, the results of both data sources analyzed in this study were qualitatively similar in that the changes in student physical activity from baseline to follow-up did not significantly differ in intervention and comparison conditions, giving confidence in the conclusions. Next, data about whether the student was enrolled in PE at the time of data collection is not available; therefore, it is not possible to determine whether student physical activity was measured in the school semester in which students were not enrolled in PE. This may be somewhat problematic as previous evidence suggests that secondary students enrolled in PE accumulate slightly greater amounts of physical activity compared to students not enrolled in PE [57]. Nevertheless, the aims of the PE policy are to enable students to achieve the recommended physical activity levels, as well as teach the necessary skills and knowledge for students to remain physically active. Therefore, it is reasonable to expect students to maintain physical activity levels throughout the school year, including the semester in which they are not enrolled in PE.

## Conclusion

Effective policies for increasing physical activity among adolescents are needed. A province-wide school-based PE policy mandating a PE credit in each of grades 9 through 12 had no effect on student physical activity overall or by student and school characteristics; however, these results should be interpreted with caution given the use of a nonrandomized research design and a lack of data assessing the extent of policy implementation in schools. Nevertheless, this study provides much needed evidence about policy features that may help to optimize the current PE policy as well as future PE policies in other jurisdictions, including the dose of physical activity required to promote recommended levels of physical activity and the need for targeted approaches in schools in low SES neighbourhoods. More research examining the effect of province-wide school PE policies on student physical activity overall, and among population sub-groups, is critical for informing intervention design and ensuring that limited resources are utilized effectively.

## Abbreviations

BMI: Body mass index
CI: Confidence interval
MIPASS: Manitoba investigating physical activity among secondary students
MVPA: Moderate to vigorous physical activity
OR: Odds ratio
PE: Physical Education
